# HSP70 drives myoblast fusion during C2C12 myogenic differentiation

**DOI:** 10.1242/bio.053918

**Published:** 2020-07-22

**Authors:** Savant S. Thakur, Kristy Swiderski, Victoria L. Chhen, Janine L. James, Nicki J. Cranna, A. M. Taufiqual Islam, James G. Ryall, Gordon S. Lynch

**Affiliations:** Centre for Muscle Research, Department of Physiology, University of Melbourne, Victoria, Australia 3010

**Keywords:** Heat shock protein 70, Myogenesis, Fusion, C2C12, Skeletal muscle

## Abstract

In response to injury, skeletal muscle stem cells (MuSCs) undergo myogenesis where they become activated, proliferate rapidly, differentiate and undergo fusion to form multinucleated myotubes. Dramatic changes in cell size, shape, metabolism and motility occur during myogenesis, which cause cellular stress and alter proteostasis. The molecular chaperone heat shock protein 70 (HSP70) maintains proteostasis by regulating protein biosynthesis and folding, facilitating transport of polypeptides across intracellular membranes and preventing stress-induced protein unfolding/aggregation. Although HSP70 overexpression can exert beneficial effects in skeletal muscle diseases and enhance skeletal muscle repair after injury, its effect on myogenesis has not been investigated. Plasmid-mediated overexpression of HSP70 did not affect the rate of C2C12 proliferation or differentiation, but the median number of myonuclei per myotube and median myotube width in differentiated C2C12 myotubes were increased with HSP70 overexpression. These findings reveal that increased HSP70 expression can promote myoblast fusion, identifying a mechanism for its therapeutic potential to enhance muscle repair after injury.

This article has an associated First Person interview with the first author of the paper.

## Abstract (precis)

**Summary:** In C2C12 myoblasts, HSP70 overexpression does not alter proliferation or differentiation but enhances fusion to increase myotube size.

## INTRODUCTION

Skeletal muscle has a remarkable ability to regenerate in response to injury due to its resident population of adult muscle stem cells (MuSCs). Following injury, MuSCs are activated, enter the cell cycle and become specified to the myogenic lineage after which they proliferate rapidly, differentiate and ultimately undergo fusion and maturation (Yin et al., 2013). During myogenesis, dramatic changes occur in cell size, shape, metabolism, and motility, which alter proteostasis and cause cellular stress (Tang and Rando, 2014). Stressed cells produce heat shock proteins (HSPs) as an adaptive mechanism to survive the insult. Heat shock protein 70 (HSP70), the most well studied member of the highly conserved HSP family of molecular chaperones, protects against cellular stress and maintains proteostasis (Clerico et al., 2015; Thakur et al., 2018).

Transgenic manipulation of HSP70 and pharmacological induction of Hsp72 (the inducible form of HSP70) have beneficial effects in muscle conditions including ageing, diabetes, obesity, disuse atrophy and Duchenne muscular dystrophy (Chung et al., 2008; Drew et al., 2014; Gehrig et al., 2012; Henstridge et al., 2014; Kennedy et al., 2016; McArdle et al., 2004; Senf et al., 2008). Furthermore, studies in HSP70^−/−^ mice identified an important role for extracellular HSP70 in muscle repair through regulating the innate immune response and the activity of endogenous MuSCs at the site of damage (Senf et al., 2013). While HSP70 has well-established roles in regulating stress in skeletal muscle, comparatively little is known of its role in myogenesis (Liu et al., 2006; Senf et al., 2013).

HSP70 expression has been detected in slow type I fibres in mouse soleus and plantaris muscles at embryonic day 22 (Ogata et al., 2003), and muscle fibres in mice with a systemic deletion of HSP70 have a reduced cross-sectional area (Senf et al., 2013). In addition, whole transcriptome analyses of quiescent and activated MuSCs and proliferating and differentiating muscle cells revealed differential expression of the genes encoding HSP70 (Liu et al., 2013; Ryall et al., 2015). More recently, studies in C2C12 cells have identified a role for HSP70 in myoblast differentiation via stabilisation of p38MAPK (Fan et al., 2018). We and others have found that protein expression of HSP70 increases during the early stages of differentiation relative to that in proliferating myoblasts (Fan et al., 2018; Thakur et al., 2019), peaking at the onset of myoblast fusion, but the consequences of increased HSP70 expression during myogenesis have not been determined. Here we have overexpressed a GFP-HSP70 fusion protein in C2C12 myoblasts and investigated effects on proliferation, differentiation and fusion.

## RESULTS

### GFP-HSP70 overexpression does not alter subcellular localisation of HSP70

**Fig. 1.**
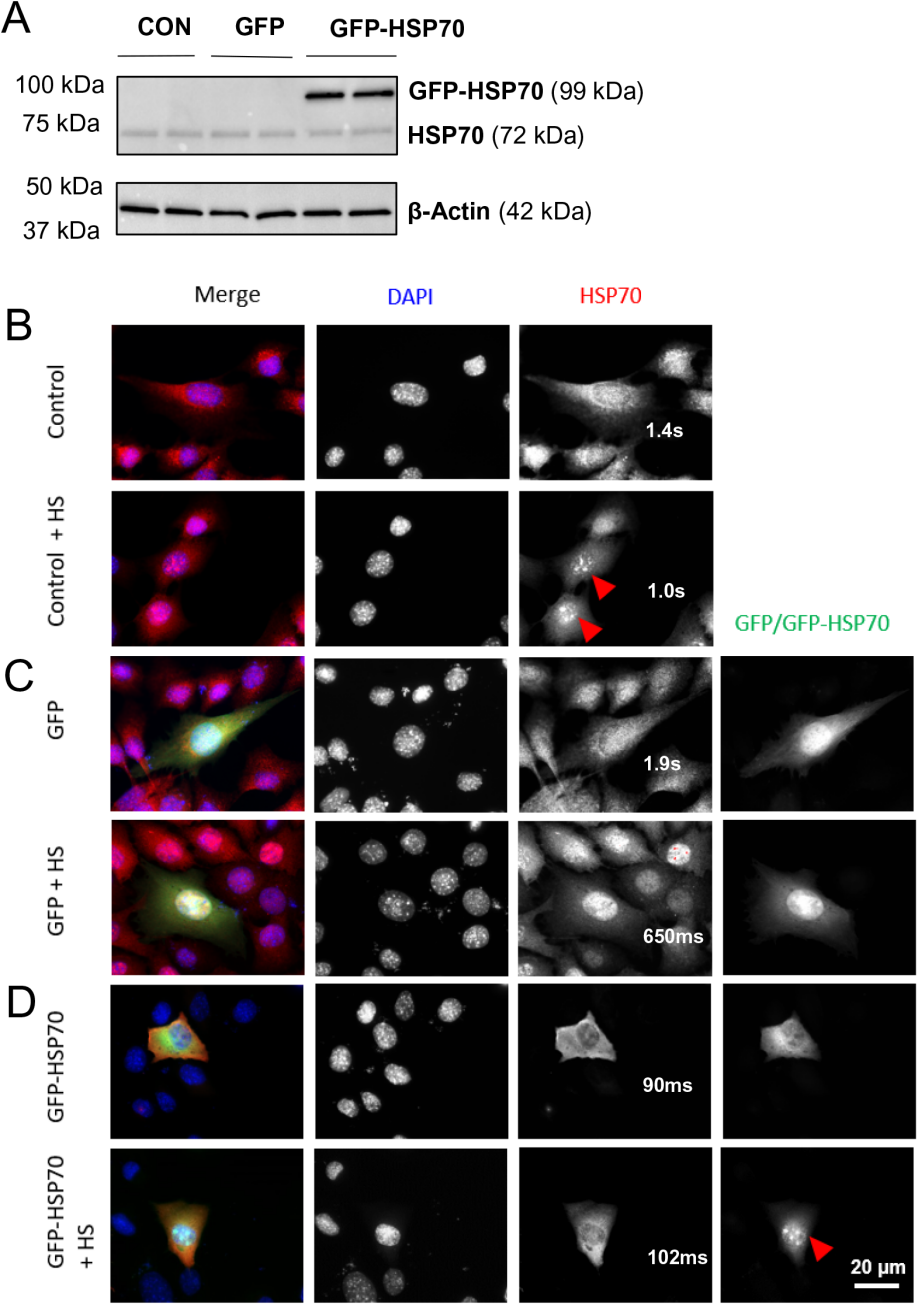
**Expression and subcellular localisation of GFP-HSP70 in C2C12 cells.** Plasmid DNA encoding either GFP or a GFP-HSP70 fusion protein was transfected into proliferating C2C12 cells and the expression and localisation of each protein was assessed. (A) Western blot showing protein expression of endogenous HSP70 and the GFP-HSP70 fusion protein in control C2C12 cells (CON; not transfected), or cells transfected with plasmids encoding GFP or GFP-HSP70. β-actin is shown as the loading control. Representative immunofluorescence images of control C2C12 cells (B) or cells transfected with GFP (C) or GFP-HSP70 (D) in control conditions (top row) or following 2 h of heat shock at 42°C (+HS, bottom row). Cells were stained with DAPI (blue) and HSP70 (red), and display GFP/GFP-HSP70 (green) signal. Exposure times are indicated.

Before assessing the effect of HSP70 overexpression on myoblast functions, we first confirmed expression and subcellular localisation of the GFP-HSP70 fusion protein. C2C12 cells were either left untransfected (control), or transfected with GFP or GFP-HSP70, incubated for 24 h, then lysed for protein extraction. Western immunoblotting of C2C12 cell lysates probed with HSP70 antibody confirmed the presence of a 99 kDa band corresponding to GFP-HSP70 (Fig. 1A). No GFP-HSP70 was detected in either untransfected (CON) or GFP-transfected cells. Endogenous HSP70 (72 kDa) was expressed at similar levels across all groups (Fig. 1A).

We next examined the subcellular localisation of HSP70 in control (Fig. 1B), GFP-transfected (Fig. 1C), and GFP-HSP70-transfected (Fig. 1D) C2C12 myoblasts under control conditions and in response to heat shock for 2 h at 42°C. Endogenous HSP70/72 localised diffusely throughout the cytoplasm and the nucleus of both control (Fig. 1B; top) and GFP-transfected (Fig. 1C; top) cells, and translocated to the nucleus after heat shock (Fig. 1B,C; bottom). In GFP-transfected C2C12 myoblasts, GFP was localised throughout the cytoplasm and nucleus and its localisation did not change after heat shock (Fig. 1C). In GFP-HSP70-transfected cells, the GFP-HSP70 transgene was present mainly in the cytoplasm under control conditions (Fig. 1D; top) but became concentrated in the nucleus after heat shock, similar to endogenous HSP70/72 (Fig. 1D; bottom). Together, these data demonstrate that the GFP-HSP70 fusion protein is expressed in transfected C2C12 cells and retains the ability of endogenous HSP70 to translocate to the nucleus after heat shock.

### GFP-HSP70 overexpression does not alter the rate of C2C12 cell proliferation or differentiation

**Fig. 2.**
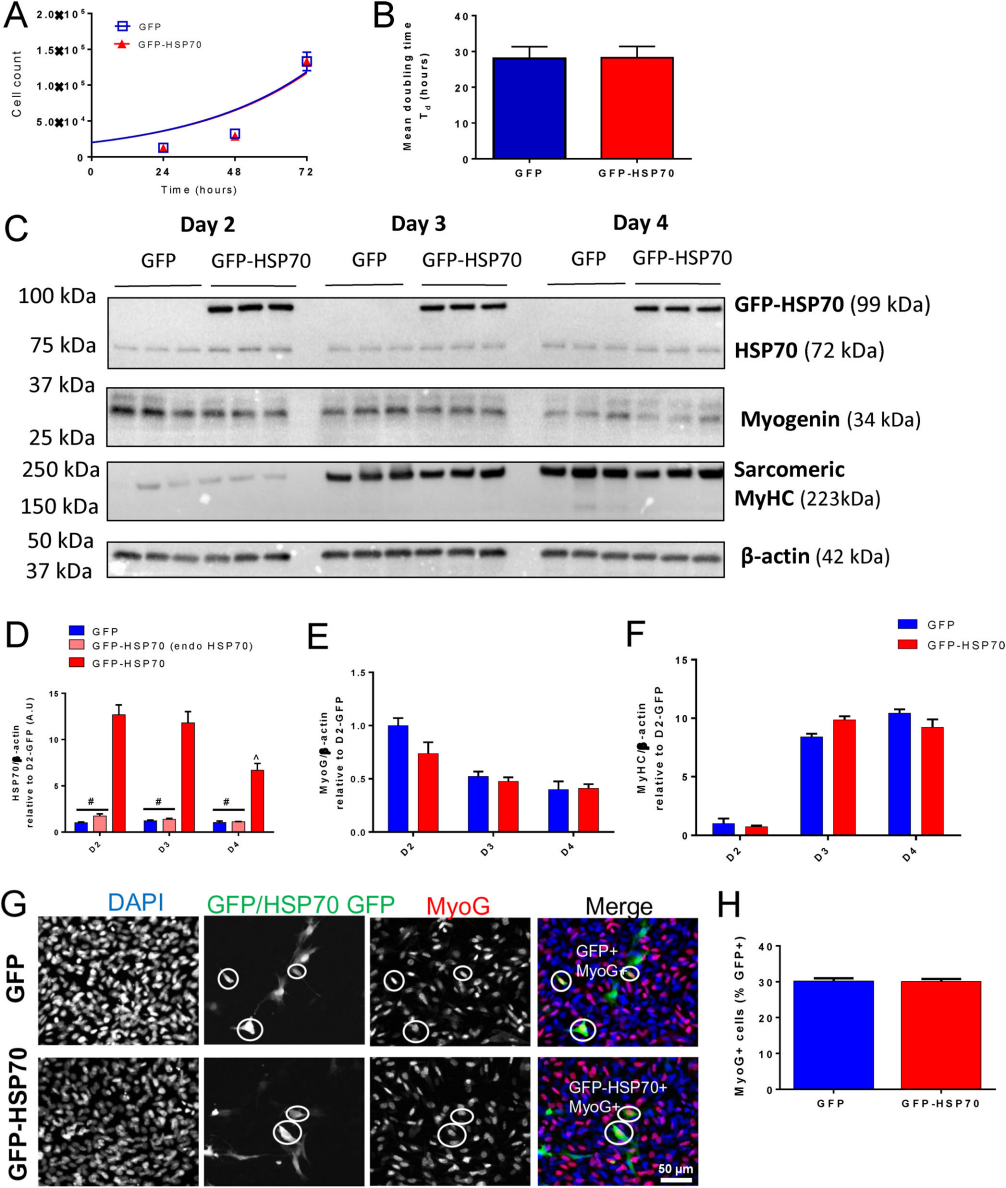
**GFP-HSP70 overexpression does not alter the rate of C2C12 cell proliferation or differentiation.** Plasmid DNA encoding either GFP or a GFP-HSP70 fusion protein were transfected into proliferating C2C12 cells and the effect on proliferation and differentiation was assessed. (A) Total cell counts at 24 h, 48 h and 72 h post-transfection were used to generate exponential growth curves for the GFP and GFP-HSP70 groups. (B) Mean doubling time (T_d_) was calculated from the exponential growth curves. Data are presented as mean±s.e.m. for the cell counts and mean±95% confidence interval for the doubling time; *n*=6 replicates/group/timepoint. (C) Representative western blots and quantification of HSP70 (D), myogenin (E), MyHC (F), relative to actin expression in C2C12 cells after 2, 3 and 4 days of differentiation. Data are presented as mean±s.e.m. and compared with a two-way ANOVA and Tukey's post-hoc test; *n*=3 replicates/group; #*P<*0.05 versus GFP-HSP70 group; ^*P<*0.05 versus D2 GFP-HSP70. (G) Representative immunofluorescence images of C2C12 cells transfected with GFP (top row) or GFP-HSP70 (bottom row) and stained with MyoG at D1. GFP+ MyoG+ cells are circled. (H) The proportion of GFP+ or GFP-HSP70+ cells stained for MyoG was determined. Data are presented as mean±s.e.m.; *n*=3 replicates/group.

To determine whether HSP70 overexpression alters C2C12 cell proliferation, cells were seeded at a low density and raw cell counts were taken at 24, 48 and 72 h post-transfection. Blue boxes representing the number of GFP transfected cells and red triangles representing GFP-HSP70 transfected cells overlapped almost completely at each timepoint and the growth curves were identical (Fig. 2A). No difference was observed in the mean doubling times of C2C12 myoblasts transfected with either GFP or GFP-HSP70 (Fig. 2B). Therefore, HSP70 overexpression does not alter the proliferative capacity of C2C12 myoblasts.

The ability of C2C12 cells transfected with either GFP or GFP-HSP70 to undergo differentiation was next assessed by western immunoblotting. Expression of endogenous HSP70 in GFP-transfected and GFP-HSP70 transfected cells was similar at D2, D3 and D4 of differentiation (Fig. 2C,D). The GFP-HSP70 fusion protein was detected in all GFP-HSP70 transfected cells resulting in a 12-, 9-, and 6-fold overexpression of HSP70 at D2, D3, and D4 of differentiation, respectively. At D4 GFP-HSP70 overexpression was decreased relative to D2 (Fig. 2C,D), consistent with previous reports of HSP70 expression levels during C2C12 differentiation (Fan et al., 2018; Thakur et al., 2019). Analysis of myogenin (Fig. 2C,E) and sarcomeric myosin heavy chain (Fig. 2C,F) protein expression showed an expected decrease in myogenin expression and concomitant increase in sarcomeric myosin heavy chain at D3 and D4 of differentiation relative to D2. No differences were detected in the expression of either myogenin (Fig. 2C,E) or sarcomeric myosin heavy chain (Fig. 2C,F) between GFP-transfected cells and GFP-HSP70-transfected cells.

To further examine the effect of HSP70 overexpression on early differentiation, myogenin immunofluorescence at D1 of differentiation was investigated. A similar proportion of GFP-transfected cells and GFP-HSP70-transfected cells expressed myogenin at this timepoint (Fig. 2G,H). In contrast to previous reports indicating HSP70 deletion impairs myoblast differentiation (Fan et al., 2018), these results show that HSP70 overexpression does not alter the ability of C2C12 cells to differentiate or their rate of differentiation.

### C2C12 cells transfected with GFP-HSP70 undergo enhanced fusion during differentiation

**Fig. 3.**
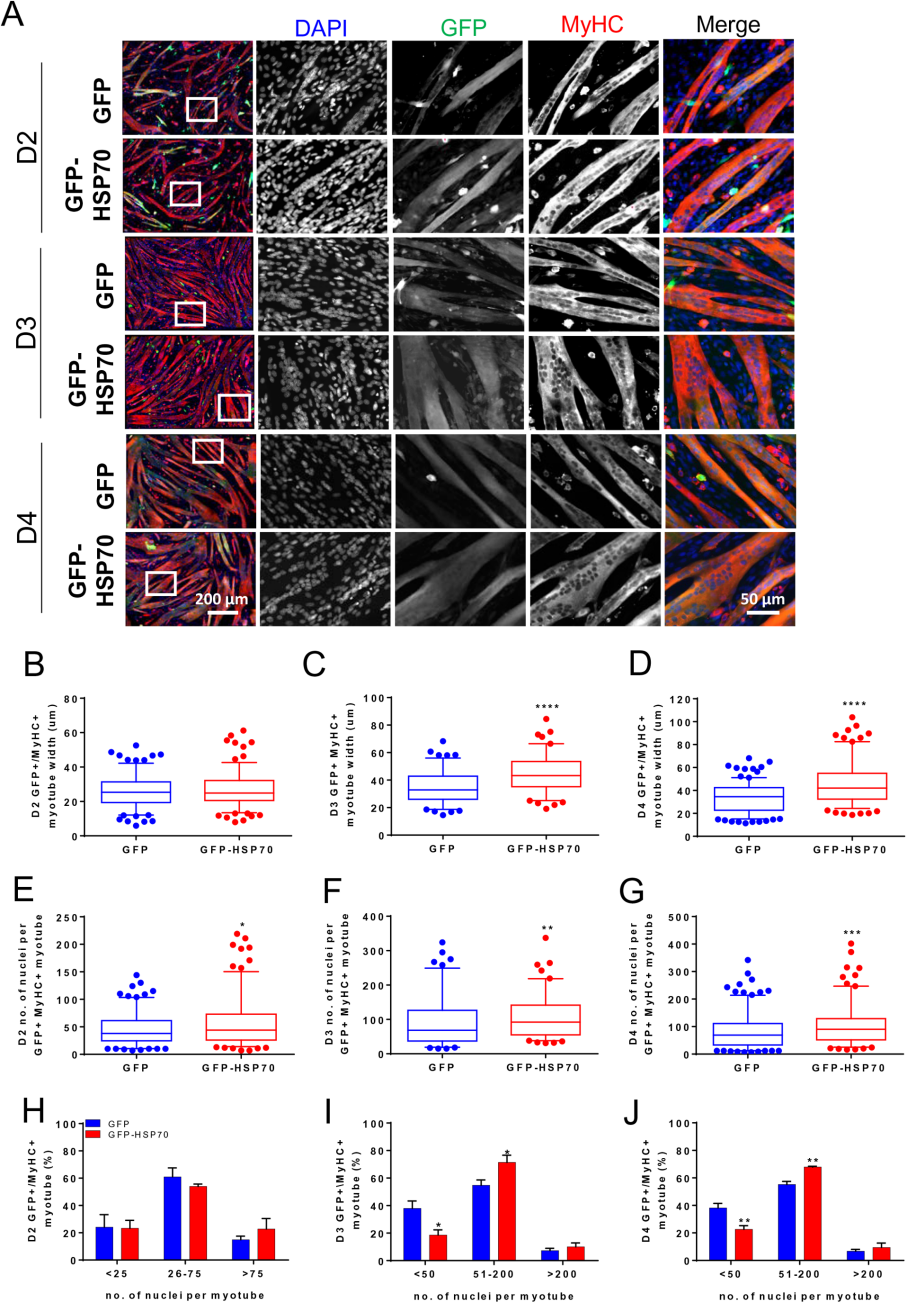
**GFP-HSP70 overexpression increases myotube nuclei number and width.** (A) C2C12 cells transfected with GFP or GFP-HSP70 were differentiated for either 2 (D2), 3 (D3) or 4 (D4) days and stained with MyHC (MF20). (A) Representative microscopic fields are shown and white-boxed regions are shown at higher magnification. Myotube diameter was measured at D2 (B), D3 (C) and D4 (D) of differentiation. The number of nuclei per myotube was counted at D2 (E), D3 (F) and D4 (G) of differentiation. Data are presented as 5th–95th percentile box and whisker plot and compared with a Mann–Whitney test; *n*>100 myotubes were analysed; *P<0.05, **P<0.01, ***P<0.001, ****P<0.0001 versus GFP. The proportion of myotubes containing less than 50, between 51 and 200, or more than 200 nuclei was determined at D2 (H), D3 (I), and D4 (J) of differentiation. Data are presented as mean±s.e.m. and compared with a two-way ANOVA and Tukey's post-hoc test; *n*>100 myotubes were analysed and averaged from three independent experiments. *P<0.05, **P<0.01 versus GFP.

As HSP70 overexpression had no impact on proliferation or differentiation of C2C12 myoblasts, we next examined the effect on myoblast fusion and myotube formation. C2C12 cells were transfected with either GFP or GFP-HSP70 and differentiated for 2, 3, or 4 days after which the size and number of nuclei per myotube was determined. The median width of myotubes was increased in GFP-HSP70 transfected cells relative to GFP-transfected cells at D3 (Fig. 3A,C) and D4 (Fig. 3A,D) but not D2 (Fig. 3A,B) of differentiation. In addition, the median number of nuclei per myotube was increased in GFP-HSP70 transfected cells relative to GFP-transfected cells at D2 (Fig. 3A,E), D3 (Fig. 3A,F) and D4 (Fig. 3A,G) of differentiation.

We next analysed the number of nuclei per myotube, which was significantly reduced in GFP-HSP70-transfected myotubes containing less than 50 myonuclei; significantly increased in myotubes with 51–200 nuclei; and unchanged in myotubes with more than 200 nuclei relative to GFP-transfected myotubes at D3 (Fig. 3I) and D4 (Fig. 3J) but not D2 (Fig. 3H) of differentiation. Together, these findings indicate that HSP70 overexpression enhances myoblast fusion to increase myotube size.

## DISCUSSION

Evidence to date supports a positive influence for HSP70 on myogenic differentiation, which is consistent with evidence from other cell lineages in which HSP70 promotes differentiation of neurons (Loones et al., 2000), erythroblasts (Ribeil et al., 2007), and mesenchymal stem cells (Li et al., 2018). HSP70 expression is higher in differentiated C2C12 myotubes relative to proliferating C2C12 myoblasts (Xiao et al., 2011). In addition, HSP70 protein levels are increased in the early stages of C2C12 cell differentiation (Fan et al., 2018; Thakur et al., 2019) and knockdown of HSP70 impairs C2C12 differentiation (Fan et al., 2018). Here we have shown, for the first time, that overexpression of GFP-HSP70 enhances C2C12 myoblast fusion to increase myotube size.

Reduced or loss of HSP70 expression has previously been shown to impair myoblast differentiation, with knockdown in C2C12 myoblasts resulting in reduced myogenin and myosin heavy chain expression (Fan et al., 2018), and absence of HSP70 during embryonic myogenesis resulting in reduced muscle fibre cross-sectional area in adult mice (Senf et al., 2013). Therefore, it might be expected that HSP70 overexpression would enhance differentiation. Importantly, HSP70 overexpression altered neither C2C12 proliferation nor early differentiation, indicating that endogenous levels of HSP70 are sufficient for maximal differentiation. In contrast, overexpression of HSP70 significantly increased the rate of myoblast fusion. It could therefore be speculated that the previously reported impairment in differentiation seen in C2C12 cells after HSP70 knockdown (Fan et al., 2018) might be due to a failure of fusion rather than a failure to initiate differentiation. In support of this, the authors report the presence of fewer nuclei in myotubes after HSP70 knockdown (Fan et al., 2018). Taken together, this suggests HSP70 plays an important role as a regulator of myoblast fusion.

While the mechanism underlying enhanced myoblast fusion after HSP70 overexpression in C2C12 cells was not investigated here, it is possible the previously identified HSP70-linked stabilisation of p38MAPK (Fan et al., 2018) facilitates signalling that drives myotube fusion. Inhibition of p38MAPK inhibits C2C12 myotube formation *in vitro* (Wu et al., 2000), an effect that can be directly attributed to a block of myotube fusion (Gardner et al., 2015), and overexpression of p38MAPK rescues impaired differentiation after HSP70 knockdown (Fan et al., 2018). In addition, HSP70 interacts directly with p38MAPK (Gong et al., 2012), and overexpression of HSP70 increases the half-life of the p38MAPK protein (Fan et al., 2018). p38MAPK has been proposed to drive myogenic differentiation via activation of MyoD, but this appears to be independent of increased myogenin or myosin heavy chain expression which did not change with HSP70 overexpression. Furthermore, whether HSP70 and/or p38MAPK alter the expression or activity of the fusogenic proteins (e.g. myomaker, myomerger) remains unknown. Therefore, exactly how HSP70-mediated stabilisation of p38MAPK promotes myoblast fusion remains to be determined.

In conclusion, HSP70 overexpression in proliferating C2C12 cells increased myotube width and the median number of myonuclei per myotube, suggesting an important role of HSP70 in driving myoblast fusion during muscle differentiation. Enhanced myoblast fusion through increased HSP70 expression supports its therapeutic potential for treating muscle injury and disorders associated with muscle atrophy.

## MATERIALS AND METHODS

### Plasmids

To examine the effect of HSP70 overexpression on C2C12 cell proliferation and differentiation, the pEGFP-C3 plasmid containing the murine HSP70 cDNA was used [pCMV-EGFP-HSP70 (GFP-HSP70)] was a gift from Lois Greene [Addgene plasmid #15215, http://n2t.net/addgene:15215; RRID:Addgene_15215; (Zeng et al., 2004)]. A plasmid vector encoding GFP [pAAV-CMV-eGFP (GFP)], a kind gift from Professor Jeffrey S. Chamberlain (Seattle, WA, USA), was used as control for overexpression experiments.

### Cell culture

Proliferating C2C12 cells (American Type Culture Collection, ATCC, Manassas, VA, USA) were cultured at 60–70% confluency in growth media [Dulbecco's Modified Eagle Medium (DMEM, 4.5 g/l D-glucose, 4.0 mM L-glutamine, no sodium pyruvate; Thermo Fisher Scientific, Waltham, MA, USA) supplemented with 10% foetal bovine serum (FBS; Thermo Fisher Scientific) and penicillin (100 units/ml)/streptomycin (100 μg/ml) (Pen/strep; Thermo Fisher Scientific)]. Cells were maintained in a humidified chamber at 37°C, in 95% air and 5% CO_2_. To induce myogenic differentiation, C2C12 cells were grown to 100% confluency and switched from growth media to low serum differentiation media [DMEM supplemented with 2% horse serum (HS; Thermo Fisher Scientific) and pen/strep]. Media was replaced with fresh differentiation media every 24 h.

### Transient transfection of C2C12 cells

One to two hours after plating, C2C12 cells were transiently transfected with plasmid DNA using Lipofectamine 3000 reagent (Thermo Fisher Scientific) according to the manufacturer's instructions. Briefly, DNA and Lipofectamine were diluted separately in Opti-MEM (Thermo Fisher Scientific). The diluted Lipofectamine was then added to DNA mixture and allowed to complex for 5 min. The resulting DNA-lipid complexes were added to the growth media and incubated for 20–22 h at 37°C in 5% CO_2_. To confirm successful transfection with pAAV-CMV-eGFP and pEGFP-HSP70 plasmids, C2C12 cells were visualised under the GFP filter of Zeiss Primovert light microscope (Carl Zeiss Pty. Ltd., Oberkochen, Baden­Württemberg, Germany) and images were acquired with Axiocam ERc5s camera using Zen software (Carl Zeiss Pty. Ltd.).

### Cell proliferation assay

To analyse the rate of C2C12 cell proliferation, 2.0×10^4^ cells were seeded per well in 1.5 ml growth media onto six-well plates (Corning Costar cell culture plates; Sigma-Aldrich, St Louis, MO, USA). Two hours after seeding, cells were transfected with 1.0 μg of either GFP (*n*=6/timepoint) or GFP-HSP70 (*n*=6/timepoint) plasmid DNA per well. The growth media was refreshed 20 h post-transfection to remove excess transfection mix. Total cell numbers were determined at 24, 48 and 72 h post-transfection using a TC20 automated cell counter (Bio-Rad Laboratories, Hercules, CA, USA), with each measurement performed in duplicate. An exponential growth curve was generated from the cell counts and used to calculate the mean doubling time (T_d_).

### Direct and indirect fluorescence

For immunofluorescence analyses, 2×10^5^ C2C12 cells were seeded per well in 1 ml growth media in 12-well plates (Corning Costar cell culture plates; Sigma-Aldrich) containing 18 mm round glass coverslips (Neuvitro, Vancouver, WA, USA). Cells were transfected with 1 μg of GFP (*n*=3/timepoint) or GFP-HSP70 (*n*=3/timepoint) plasmid DNA per well, 1 h post-seeding. Once the cells reached 100% confluency, the growth media was replaced with differentiation media to induce myogenic differentiation. The cells were then allowed to differentiate for 1, 2, or 3 days with media changes every 24 h. At 1, 2, or 3 days post-differentiation, cells were fixed with 4% paraformaldehyde (PFA; Aesar, Ward Hill, MA, USA) in phosphate buffered saline (PBS; Thermo Fisher Scientific) for 15 min, washed in PBS once and then permeablised with 0.1% Triton X-100 (Sigma-Aldrich) in PBS for 10 min. Non-specific binding sites were blocked with 3% bovine serum albumin (BSA, Sigma-Aldrich) in PBS for 45 min at room temperature. Target proteins were detected by incubating coverslips in blocking solution containing primary antibodies against mouse-α-myogenin IgG1 (1:200; MA5-11486; Thermo Fisher Scientific) and mouse-α-sarcomeric myosin heavy chain IgG2b [1:50; deposited to the University of Iowa Developmental Studies Hybridoma Bank by Fischman, D.A. (DSHB Hybridoma Product MF 20)] at 4°C overnight. On the following day, coverslips were washed in PBS (3×5 min) and incubated in blocking solution containing Alexa Fluor 555-conjugated goat-α-mouse IgG1 (1:1000; A21127; Thermo Fisher Scientific) and Alexa Fluor 555-conjugated goat-α-mouse IgG2b (1:1000; A21147; Thermo Fisher Scientific) for 2 h at room temperature in the dark. The coverslips were then washed with PBS (3×5 min) and counterstained with 4′6-diamidino-2-phenylindole (DAPI; 1:1000, Thermo Fisher Scientific) in PBS. Finally, coverslips were mounted onto glass slides using a water based mounting medium (Fluoro-Gel; ProSciTech Pty. Ltd., Kirwan, QLD, Australia) and coated in nail polish.

Immunofluorescence images were acquired on a Zeiss Axio Imager M2 microscope with a monochrome camera (AxioCam 506, Carl Zeiss Pty. Ltd.) using Zen software (Carl Zeiss Pty. Ltd.). Images were taken for Texas red, GFP and DAPI channels. Image analysis was performed using ImageJ software (National Institute of Health, NIH, Bethesda, MD, USA). The number of GFP or GFP-HSP70 transfected cells that stained positively for MyoG were counted for each field of the view at days 1 and 2 and expressed as a percentage of the total number of GFP or GFP-HSP70+ cells. This proportion was used as an index of the early differentiation rate. Myotube width was quantified at day 3 post-differentiation by calculating the mean of three measurements along each the length of each GFP or GFP-HSP70+ myotube stained with MyHC. The number of myonuclei present in each GFP or GFP-HSP70 transfected myotube were also determined and used as index of cell fusion.

### Western immunoblotting

Cells were washed twice in 2 ml PBS per well, PBS was aspirated from the wells and intact six-well plates were stored at −80°C until the day of lysis and protein extraction. Whole cell lysates were prepared as described previously (Park et al., 2016). Briefly, cells were washed with ice-cold PBS containing phenylmethylsulfonyl fluoride (PMSF, 1 mM, Thermo Fisher Scientific) and subsequently lysed in ice-cold radioimmunoprecipitation assay (RIPA) lysis buffer [50 mM TrisHCl (pH 7.4), 150 mM NaCl, 1 mM EDTA, 1% Triton X-100, 1% Na-Deoxycholic acid and 0.1% sodium dodecyl sulphate (SDS); Millipore, Billerica, MA, USA] containing protease inhibitor cocktail (PIC, 1 mM, Sigma-Aldrich) using a cell scraper (Corning Costar; Sigma-Aldrich). Cell lysates were sonicated for 15 s using a Microson XL-2000 sonicator (Misonix, NY, USA) to disrupt the nuclear membrane, centrifuged for 10 min at 10,000 rpm and 4°C to remove cellular debris, and stored at −80°C until further analysis. Total protein concentration was determined through a DC protein assay (Bio-Rad Laboratories) by measuring absorbance at 750 nm using a Multiskan Spectrum spectrophotometer (Thermo Fisher Scientific). All samples were equalised to a protein concentration of 1 μg/μl with RIPA lysis buffer. Laemmli buffer [4×; 0.25 M TrisHCl (pH 6.8), 6% SDS, 40% glycerol, 0.04% bromophenol blue, 16% Dithiothreitol (DTT)] was then added and samples were incubated for 5 min at 95°C. Identical amounts of each sample (10 μg) were loaded onto pre-cast SDS-polyacrylamide gels (4–15% Criterion TGX Stain-Free Precast Gels, Bio-Rad Laboratories) in Tris/Glycine/SDS (TGS) buffer (Bio-Rad Laboratories) and resolved using SDS-PAGE for 45 min at 200 V. Proteins were transferred onto polyvinylidene difluoride (PVDF) membranes using a Trans-blot Turbo Blotting System (Bio-Rad Laboratories) by applying a constant current (1 amp) for 7 min. Efficient protein transfer was confirmed using post-transfer stain free image of gel and membrane. Non-specific binding sites were blocked by incubating the PVDF membrane in 3% BSA in Tris-buffered saline containing Tween 20 (TBST; 50 mM TrisHCl, 150 mM NaCl, 0.05% Tween 20) for 2 h at room temperature. Target proteins were detected by probing the PVDF membrane with either rabbit-α-HSP70 (1:5000; ADI-SPA-812, Enzo Life Science, Farmingdale, NY, USA), mouse-α-myogenin (1:400; sc12732, Santa Cruz Biotechnology, Rockford, IL, USA), mouse-α-MF20 (1:5000; DSHB Hybridoma Product MF 20), or rabbit-α-β-actin (1:1000; Cell Signaling Technology, Danvers, MA, USA) diluted in 3% BSA/TBST overnight at 4°C. The following day, PVDF membrane was washed in TBST (1×2 min, 3×10 min) and incubated with 3% BSA/TBST containing either HRP-sheep-α-mouse or HRP-donkey-α-rabbit IgG (1:5000; GE Life Sciences, Buckinghamshire, UK) for 1 h at room temperature. PVDF membrane was then washed in TBST (1×2 min, 3×10 min) and immunodetection was performed using Supersignal West Femto Chemiluminescent Substrate (Thermo Fisher Scientific). The membrane was visualised and imaged on a Chemidoc–System (Bio-Rad Laboratories). The protein bands were analysed using ImageLab software (Bio-Rad Laboratories) and the intensity band of interest was normalised to total protein.

### Statistical analysis

Data are presented as mean±s.e.m. unless indicated otherwise. Unpaired Student's *t*-test was used to compare differences between GFP and GFP-HSP70 groups. When the assumption of Gaussian distribution was not met, a non-parametric Mann–Whitney *U*-test was used for comparisons. For comparisons between more than two groups, a one- or two-way ANOVA was performed with a Tukey's post-hoc test used when statistical differences were detected. The level of significance was set at *P*<0.05 for all comparisons.

## References

[BIO053918C1] Chung J., Nguyen A.-K., Henstridge D. C., Holmes A. G., Chan M. H. S., Mesa J. L., Lancaster G. I., Southgate R. J., Bruce C. R., Duffy S. J. et al. (2008). Hsp72 protects against obesity-induced insulin resistance. *Proc. Natl. Acad. Sci. USA* 105, 1739-1744. 10.1073/pnas.0705799105 18223156 PMC2234214

[BIO053918C2] Clerico E. M., Tilitsky J. M., Meng W. and Gierasch L. M. (2015). How Hsp70 molecular machines interact with their substrates to mediate diverse physiological functions. *J. Mol. Biol.* 427, 1575-1588. 10.1016/j.jmb.2015.02.004 25683596 PMC4440321

[BIO053918C3] Drew B. G., Ribas V., Le J. A., Henstridge D. C., Phun J., Zhou Z., Soleymani T., Daraei P., Sitz D., Vergnes L. et al. (2014). Hsp72 is a mitochondrial stress sensor critical for parkin action, oxidative metabolism, and insulin sensitivity in skeletal muscle. *Diabetes* 63, 1488-1505. 10.2337/db13-0665 24379352 PMC3994950

[BIO053918C4] Fan W., Gao X. K., Rao X. S., Shi Y. P., Liu X. C., Wang F. Y., Liu Y. F., Cong X. X., He M. Y., Xu S. B. et al. (2018). Hsp70 interacts with mitogen-activated protein kinase (Mapk)-activated protein kinase 2 to regulate P38mapk stability and myoblast differentiation during skeletal muscle Regeneration. *Mol. Cell. Biol.* 38, e00211-e00218. 10.1128/mcb.00211-18 30275345 PMC6275188

[BIO053918C5] Gardner S., Gross S. M., David L. L., Klimek J. E. and Rotwein P. (2015). Separating myoblast differentiation from muscle cell fusion using Igf-I and the P38 map kinase inhibitor Sb202190. *Am. J. Physiol. Cell Physiol.* 309, C491-C500. 10.1152/ajpcell.00184.2015 26246429 PMC4593770

[BIO053918C6] Gehrig S. M., Van Der Poel C., Sayer T. A., Schertzer J. D., Henstridge D. C., Church J. E., Lamon S., Russell A. P., Davies K. E., Febbraio M. A. et al. (2012). Hsp72 preserves muscle function and slows progression of severe muscular dystrophy. *Nature* 484, 394-398. 10.1038/nature10980 22495301

[BIO053918C7] Gong X., Luo T., Deng P., Liu Z., Xiu J., Shi H. and Jiang Y. (2012). Stress-induced interaction between P38 Mapk and Hsp70. *Biochem. Biophys. Res. Commun.* 425, 357-362. 10.1016/j.bbrc.2012.07.096 22842575

[BIO053918C8] Henstridge D. C., Bruce C. R., Drew B. G., Tory K., Kolonics A., Estevez E., Chung J., Watson N., Gardner T., Lee-Young R. S. et al. (2014). Activating Hsp72 in rodent skeletal muscle increases mitochondrial number and oxidative capacity and decreases insulin resistance. *Diabetes* 63, 1881-1894. 10.2337/db13-0967 24430435 PMC4030108

[BIO053918C9] Kennedy T. L., Swiderski K., Murphy K. T., Gehrig S. M., Curl C. L., Chandramouli C., Febbraio M. A., Delbridge L. M. D., Koopman R. and Lynch G. S. (2016). Bgp-15 improves aspects of the dystrophic pathology in Mdx and Dko mice with differing efficacies in heart and skeletal muscle. *Am. J. Pathol.* 186, 3246-3260. 10.1016/j.ajpath.2016.08.008 27750047

[BIO053918C10] Li C., Sunderic K., Nicoll S. B. and Wang S. (2018). Downregulation of heat shock protein 70 impairs osteogenic and chondrogenic differentiation in human mesenchymal stem cells. *Sci. Rep.* 8, 553 10.1038/s41598-017-18541-1 29323151 PMC5765044

[BIO053918C11] Liu Y., Gampert L., Nething K. and Steinacker J. M. (2006). Response and function of skeletal muscle heat shock protein 70. *Front. Biosci.* 11, 2802-2827. 10.2741/2011 16720354

[BIO053918C12] Liu L., Cheung T. H., Charville G. W., Hurgo B. M. C., Leavitt T., Shih J., Brunet A. and Rando T. A. (2013). Chromatin modifications as determinants of muscle stem cell quiescence and chronological aging. *Cell Rep.* 4, 189-204. 10.1016/j.celrep.2013.05.043 23810552 PMC4103025

[BIO053918C13] Loones M.-T., Chang Y. H. and Morange M. (2000). The distribution of heat shock proteins in the nervous system of the unstressed mouse embryo suggests a role in neuronal and non-neuronal differentiation. *Cell Stress Chaperones* 5, 291-305. 10.1379/1466-1268(2000)005<0291:TDOHSP>2.0.CO;2 11048652 PMC312859

[BIO053918C14] Mcardle A., Dillmann W. H., Mestril R., Faulkner J. A. and Jackson M. J. (2004). Overexpression of Hsp70 in mouse skeletal muscle protects against muscle damage and age-related muscle dysfunction. *FASEB J.* 18, 355-357. 10.1096/fj.03-0395fje 14688209

[BIO053918C15] Ogata T., Oishi Y., Roy R. R. and Ohmori H. (2003). Endogenous expression and developmental changes of Hsp72 in rat skeletal muscles. *J. Appl. Physiol.* 95, 1279-1286. 10.1152/japplphysiol.00353.2003 12909603

[BIO053918C16] Park S.-Y., Yun Y., Lim J.-S., Kim M.-J., Kim S.-Y., Kim J.-E. and Kim I.-S. (2016). Stabilin-2 modulates the efficiency of myoblast fusion during myogenic differentiation and muscle regeneration. *Nat. Commun.* 7, 10871 10.1038/ncomms10871 26972991 PMC4793076

[BIO053918C17] Ribeil J.-A., Zermati Y., Vandekerckhove J., Cathelin S., Kersual J., Dussiot M., Coulon S., Moura I. C., Zeuner A., Kirkegaard-Sørensen T. et al. (2007). Hsp70 regulates erythropoiesis by preventing Caspase-3-mediated cleavage of Gata-1. *Nature* 445, 102-105. 10.1038/nature05378 17167422

[BIO053918C18] Ryall J. G., Dell'orso S., Derfoul A., Juan A., Zare H., Feng X., Clermont D., Koulnis M., Gutierrez-Cruz G., Fulco M. et al. (2015). The Nad^+^-dependent Sirt1 deacetylase translates a metabolic switch into regulatory epigenetics in skeletal muscle stem cells. *Cell Stem Cell* 16, 171-183. 10.1016/j.stem.2014.12.004 25600643 PMC4320668

[BIO053918C19] Senf S. M., Dodd S. L., Mcclung J. M. and Judge A. R. (2008). Hsp70 overexpression inhibits Nf-kappab and Foxo3a transcriptional activities and prevents skeletal muscle atrophy. *FASEB J.* 22, 3836-3845. 10.1096/fj.08-110163 18644837 PMC6137947

[BIO053918C20] Senf S. M., Howard T. M., Ahn B., Ferreira L. F. and Judge A. R. (2013). Loss of the inducible Hsp70 delays the inflammatory response to skeletal muscle injury and severely impairs muscle regeneration. *PLoS ONE* 8, e62687 10.1371/journal.pone.0062687 23626847 PMC3633856

[BIO053918C21] Tang A. H. and Rando T. A. (2014). Induction of autophagy supports the bioenergetic demands of quiescent muscle stem cell activation. *EMBO J.* 33, 2782-2797. 10.15252/embj.201488278 25316028 PMC4282556

[BIO053918C22] Thakur S. S., Swiderski K., Ryall J. G. and Lynch G. S. (2018). Therapeutic potential of heat shock protein induction for muscular dystrophy and other muscle wasting conditions. *Phil. Trans. R. Soc. B* 373, 20160528 10.1098/rstb.2016.0528 29203713 PMC5717528

[BIO053918C23] Thakur S. S., James J. L., Cranna N. J., Chhen V. L., Swiderski K., Ryall J. G. and Lynch G. S. (2019). Expression and localization of heat-shock proteins during skeletal muscle cell proliferation and differentiation and the impact of heat stress. *Cell Stress Chaperones* 24, 749-761. 10.1007/s12192-019-01001-2 31098840 PMC6657410

[BIO053918C24] Wu Z., Woodring P. J., Bhakta K. S., Tamura K., Wen F., Feramisco J. R., Karin M., Wang J. Y. J. and Puri P. L. (2000). P38 and extracellular signal-regulated kinases regulate the myogenic program at multiple steps. *Mol. Cell. Biol.* 20, 3951-3964. 10.1128/MCB.20.11.3951-3964.2000 10805738 PMC85749

[BIO053918C25] Xiao R., Ferry A. L. and Dupont-Versteegden E. E. (2011). Cell death-resistance of differentiated myotubes is associated with enhanced anti-apoptotic mechanisms compared to myoblasts. *Apoptosis* 16, 221-234. 10.1007/s10495-010-0566-9 21161388 PMC3045653

[BIO053918C26] Yin H., Price F. and Rudnicki M. A. (2013). Satellite cells and the muscle stem cell niche. *Physiol. Rev.* 93, 23-67. 10.1152/physrev.00043.2011 23303905 PMC4073943

[BIO053918C27] Zeng X.-C., Bhasin S., Wu X., Lee J. G., Maffi S., Nichols C. J., Lee K. J., Taylor J. P., Greene L. E. and Eisenberg E. (2004). Hsp70 dynamics in vivo: effect of heat shock and protein aggregation. *J. Cell Sci.* 117, 4991-5000. 10.1242/jcs.01373 15367583

